# Transcriptomic Profiling of Buffalo Spermatozoa Reveals Dysregulation of Functionally Relevant mRNAs in Low-Fertile Bulls

**DOI:** 10.3389/fvets.2020.609518

**Published:** 2021-01-11

**Authors:** Nilendu Paul, Arumugam Kumaresan, Mohua Das Gupta, Pradeep Nag, Pushpa Rani Guvvala, Channareddi Kuntareddi, Ankur Sharma, Sellappan Selvaraju, Tirtha Kumar Datta

**Affiliations:** ^1^Theriogenology Laboratory, Veterinary Gynaecology and Obstetrics, Southern Regional Station of ICAR – National Dairy Research Institute, Bengaluru, India; ^2^Reproductive Physiology Laboratory, ICAR – National Institute of Animal Nutrition and Physiology, Bengaluru, India; ^3^Animal Genomics Laboratory, ICAR – National Dairy Research Institute, Karnal, India

**Keywords:** buffalo bulls, spermatozoa, global transcriptome, microarray analysis, male fertility, differential gene expression

## Abstract

Although, it is known that spermatozoa harbor a variety of RNAs that may influence embryonic development, little is understood about sperm transcriptomic differences in relation to fertility, especially in buffaloes. In the present study, we compared the differences in sperm functional attributes and transcriptomic profile between high- and low-fertile buffalo bulls. Sperm membrane and acrosomal integrity were lower (*P* < 0.05), while protamine deficiency and lipid peroxidation were higher (*P* < 0.05) in low- compared to high-fertile bulls. Transcriptomic analysis using mRNA microarray technology detected a total of 51,282 transcripts in buffalo spermatozoa, of which 4,050 transcripts were differentially expressed, and 709 transcripts were found to be significantly dysregulated (*P* < 0.05 and fold change >1) between high- and low-fertile bulls. Majority of the dysregulated transcripts were related to binding activity, transcription, translation, and metabolic processes with primary localization in the cell nucleus, nucleoplasm, and in cytosol. Pathways related to MAPK signaling, ribosome pathway, and oxidative phosphorylation were dysregulated in low-fertile bull spermatozoa. Using bioinformatics analysis, we observed that several genes related to sperm functional attributes were significantly downregulated in low-fertile bull spermatozoa. Validation of the results of microarray analysis was carried out using real-time qPCR expression analysis of selected genes (*YBX1, ORAI3*, and *TFAP2C)*. The relative expression of these genes followed the same trend in both the techniques. Collectively, this is the first study to report the transcriptomic profile of buffalo spermatozoa and to demonstrate the dysregulation of functionally relevant transcripts in low-fertile bull spermatozoa. The results of the present study open up new avenues for understanding the etiology for poor fertility in buffalo bulls and to identify fertility biomarkers.

## Introduction

Buffaloes contribute a major share to the world's milk supply, and recent trends show that the volume of buffalo milk is increasing steadily at about 3% per year (1). The full production potential buffaloes, however, remain unexploited due to poor reproductive efficiency (2). Poor conception rates with artificial insemination are considered as a prime reason for low reproductive efficiency in buffaloes (1, 3). Although male and female both contribute to the success or failure of conception, the importance of using semen from high-fertile bulls for artificial breeding assumes much significance because semen from a single male is used to cover several thousands of females. Several studies tried to identify the most important sperm phenotypic characteristics associated with sperm-fertilizing potential (4, 5); however discriminating low-fertile bulls from high-fertile bulls remains to continue as a challenge. Therefore, advanced semen quality assessment techniques are needed to achieve an accurate diagnosis of bull fertility (6). Recent evidences indicate existence of specific differences in sperm functional attributes and sperm molecules such as mRNAs (7), proteins (8), and metabolites (9) between high- and low-fertile bulls.

Ejaculated spermatozoa were once considered to just transfer the paternal haploid genome into oocyte and are transcriptionally and translationally inactive (10, 11). However, recent evidences indicate that ejaculated spermatozoa retain a complex, yet specific, population of RNAs, which may have important roles in sperm development, chromatin repackaging, zygote development, and fertility (12). Accumulating evidences over a period of time indicate that sperm mRNAs might influence the fertilizing potential of spermatozoa and embryonic development (13, 14), which developed curiosity among researchers to understand the role of specific mRNAs in male fertility. Earlier studies conducted on this aspect, assessed the expression levels of a few genes in spermatozoa derived from high- and low-fertile cattle bulls and reported their relationship with bull fertility (15–18); however, the results were not always consistent. A comprehensive analysis of transcript profile of spermatozoa from bulls with defined fertility is required to identify fertility-associated genes. Recent developments in DNA microarray and next-generation sequencing techniques offer immense scope to understand global transcriptome profile of spermatozoa (14, 19). High-throughput microarray technology has been successfully used for global gene expression profiling in spermatozoa of different species (20, 21). Although global transcriptomic profile of spermatozoa and sperm transcriptomic differences between high- and low-fertile cattle bulls are available (7, 22), such information is not available for buffaloes.

With this backdrop, the present study was undertaken to establish global transcriptomic profile of buffalo spermatozoa and to identify the sperm functional and transcriptomic differences between high- and low-fertile buffalo bulls. We hypothesized that differences exist at sperm transcriptomic level between high- and low-fertile buffalo bulls, and profiling of such transcript “fingerprints” of spermatozoa collected from high- and low-fertile bulls, would help in developing tools for fertility prediction of buffalo bulls, as it is reported earlier in the case of human beings (23).

## Materials and Methods

### Ethical Statement

All the experiments were duly approved and performed in accordance with the guidelines and regulations by the Institute Animal Ethics Committee (CPCSEA/IAEC/LA/SRS-ICAR-NDRI-2017/No.11).

### Fertility Classification of Bulls

Field conception rates of a total of 21 Murrah buffalo bulls were evaluated based on a minimum of 100 inseminations per bull. Conception rate was calculated based on the number of animals conceived out of the total number of animals inseminated (up to three inseminations). The effect of non-genetic factors on conception rate was studied using least squares analysis of variance for unequal and non-orthogonal data as described previously (4). The adjusted conception rate was used for the calculation of bull fertility. Bulls with conception rate mean + 1 standard deviation were considered as high fertile (*n* = 4) and mean – 1 standard deviation was considered as low fertile (*n* = 4), while the bulls in between formed the medium fertile group (*n* = 13). The details of the conception rates of the bulls used in the study are shown in Supplementary Figure 1.

### Sperm Functional Characteristics Assessment

Frozen semen straws prepared from three ejaculates per bull were used for sperm function assays (three replicates per bull). Frozen semen straws were thawed at 37°C for 30 s before performing different sperm function assays. A minimum of 200 spermatozoa were examined in each assay, and different patterns of spermatozoa were calculated.

### Sperm Motility

Sperm motility was evaluated at 400X magnification based on the visual estimation of the percentage of sperm possessing progressive motility, and the percentage was rounded to the nearest 5%.

### Sperm Membrane Integrity

Sperm membrane integrity was assessed using carboxyfluorescein diacetate-propidium iodide (CFDA-PI; Sigma Aldrich, Germany) staining as described previously (4). Briefly, thawed semen sample was washed twice using sperm-TALP. Initially, 5 μl of CFDA (0.5 mg/ml) was added to 10 million spermatozoa and incubated at 37°C for 15 min under dark conditions. Then, 2 μl of PI (0.3 mg/ml) was added and incubated further for 2 min. After centrifugation at 800 × *g* for 3 min, the supernatant was discarded, and a thin smear was made out of the pellet. An antifading agent 1,4-diazabicyclo [2.2.2] octane (DABCO) was added, and a coverslip was applied over the smear. Spermatozoa were evaluated by fluorescent microscopy (Nikon ECLIPSE Ti-s, Japan) using FITC and TRITC filters at 400X magnification. Images from the two filters were merged to obtain a final image. Membrane intact sperm exhibited bright green fluorescence, while dead spermatozoa exhibited bright red fluorescence. Moribund spermatozoa had a mixture of green and red fluorescence.

### Sperm Acrosome Status Assessment

The fluorescein isothiocyanate-conjugated peanut agglutinin (FITC-PNA; Sigma Aldrich, Germany) staining was carried out for assessing acrosome status of spermatozoa according to the method described by Singh et al. (4). Briefly, to 10 million spermatozoa, 5 μl of FITC-PNA (0.025 mg/ml) was added and incubated at 37°C for 15 min in dark condition. After incubation, 1 μl of PI was added, and incubation was done for 2 min. Sperm-TALP (100 μl) was then added, and the samples were centrifuged at 800 × *g* for 3 min. A thin smear was made from the pellet after discarding the supernatant. An antifading agent (DABCO), was added over the dried smear to reduce fluorescence quenching. Spermatozoa were evaluated by fluorescent microscopy using FITC and TRITC filters at 400X magnification. Images from the two filters were merged to obtain a final image.

### Sperm Protamine Deficiency

Chromomycin A_3_ (CMA_3;_ Sigma Aldrich, Germany) was used to detect the protamine deficiency in spermatozoa as described previously (24) with minor modifications. A thin smear of spermatozoa was made on a clean, grease-free microscopic glass slide and air dried. The air dried smears were fixed with methanol–acetic acid (3:1 = 30 ml methanol + 10 ml acetic acid) for 5 min followed by washing with PBS to remove excessive fixative and air dried. To this, 100 μl of CMA_3_ staining solution (0.25 mg/ml CMA_3_ in Mcllvaines buffer; 7 ml of 0.1 M citric acid + 32.9 ml of 0.2 M Na_2_HPO4 7 H_2_O, pH 7.0 containing 10 mM MgCl_2_) was added and incubated for 20 min, air dried, and washed with Mcllvaines buffer to wash out excess stain. The stained smear after air drying was covered with antifading agent (DABCO), and a cover slip was overlaid. Spermatozoa were observed at 400X magnification using UV filter of a fluorescent microscope (DP71, Olympus, Japan).

### Assessment of Sperm Lipid Peroxidation

4,4-Difluoro-4-bora-3a, 4a-diaza-s-indacene (BODIPY; Thermo-Scientific, USA), a member of organoboron fluorescent dye, was used to assess the plasma membrane lipid peroxidation status of spermatozoa population as described previously (25) with minor modifications. Cryopreserved spermatozoa were thawed and washed twice in 1.5 ml Hepes-buffered Tyrode (HBT) buffer by centrifugation at 800° × *g* for 5 min. Ten microliters of BODIPY (2 μM) was added to 10 million spermatozoa under dark conditions and incubated at 37°C for 30 min. Subsequently, a 100-μl sperm-TALP was added and centrifuged at 800 × *g* for 3 min. Then the supernatant was discarded, and a thin smear was made out of the pellet. After adding the antifading agent (DABCO) to the smear and applying cover slip, the spermatozoa were observed under fluorescent microscope using FITC and TRITC filters. The images from both the filters were merged to obtain the final image.

**Figure 1:**
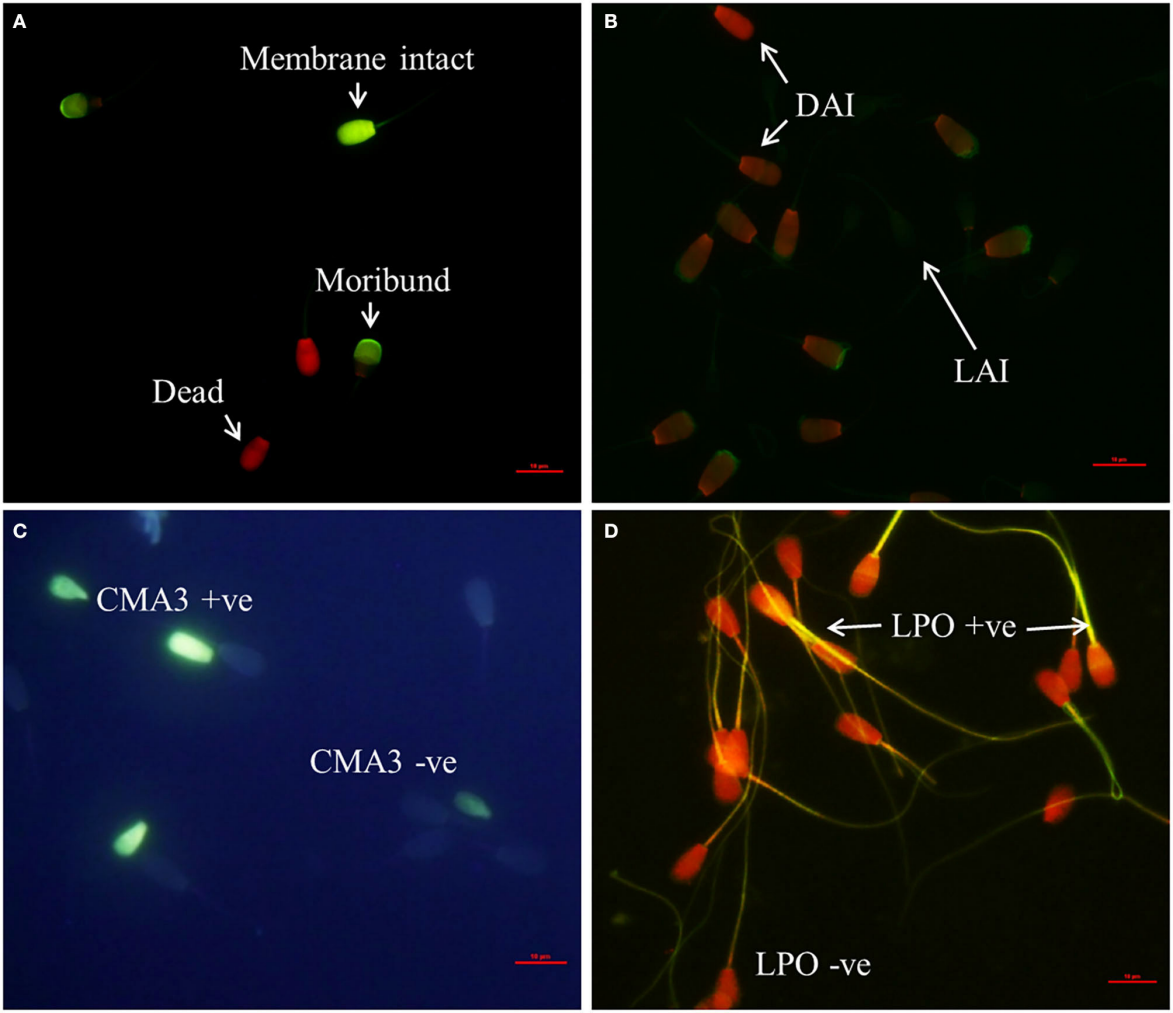
Assessment of sperm phenotypic characteristics using specific fluorochromes (400X Magnification). **(A)** CFDA-PI staining for detection of sperm membrane integrity, **(B)** FITC-PNA staining for sperm acrosome status assessment (LAI, live acrosome intact; DAI, dead acrosome intact; DAR dead acrosome reacted). **(C)** Detection of protamine deficiency in spermatozoa using Chromomycin A3 staining (CMA3 + ve, protamine deficient spermatozoa; CMA3 – ve, normal spermatozoa), **(D)** BODIPY assay for detection of sperm lipid peroxidation status (LPO + ve, lipid peroxidized spermatozoa; LPO – ve, normal spermatozoa).

The representative images for each of the above cited sperm quality analysis tests are shown in Figure 1.

### RNA Isolation, Evaluation, and Expression Microarray Hybridization

Total RNA was isolated from Percoll-selected spermatozoa using RNAeasy minikit (Qiagen, Germany) and RNA quality was assessed using bioanalyzer. Samples with optimal purity (OD 260/280 >1.8 and <2.2 and optimal concentration >50 mg/ml) were selected for use in microarray. Two representative RNA samples were made from the four high-fertile bulls by pooling equal quantities of sperm RNA from two high-fertile bulls each. Similarly, two representative RNA samples were made for low-fertile bulls. Thus, four samples (high- and low-fertile each two samples) were subjected to microarray analysis. Agilent Bos Taurus GXP 8X60k AMADID: 29411 slide formats were used for Microarray experiments (Genotypic Technology, Bengaluru, India). Briefly, the samples were labeled using the Agilent Quick-Amp labeling kit and T7 promoter-based linear amplification was used to generate labeled complementary RNA. The labeled cRNA with specific activity (>8) was hybridized on the array using the Gene Expression Hybridzation kit in Sure hybridization chambers at 65°C for 16 h. Hybridized slides were washed using wash buffers, and microarray chip signal was scanned and processed by Agilent Microarray Scanner (Agilent Technologies, United States). Images were quantified using Feature Extraction Software (Agilent). Normalization of the data was done in GeneSpring GX 12.6 using the 75th percentile shift, and the gProcessed signal (dye normalized background subtracted signal intensity) was log transformed (ID_REF = VALUE = Log base 2).

### Validation of Microarray Data Using qPCR

Transcriptional abundance of selected genes was validated in nine bulls (three each in high-, medium-, and low-fertile category). Total RNA was isolated from spermatozoa using TRIzol (Ambion, Thermo-Scientific, USA) as described previously (26) with minor modifications. In brief, cryopreserved spermatozoa (60 million/bull) was centrifuged (4,000 g for 30 min), sperm pellet was mixed well with 1 ml of somatic cell lysis buffer (0.1% SDS, 0.5% Triton X-100 in DEPC-treated water) and kept over ice for 30 min. The lysate was again centrifuged for 30 min (4,000 g at 4°C); pre-warmed TRIzol reagent (1 ml; 62°C) was added to the washed sperm pellet and shredded by passing through a 24 G needle attached to a 5-ml syringe 8 to 10 times and incubated for 10–15 min following which 200 μl of chloroform was added and mixed for 20 s and further incubated at room temperature for 15 min. The mixture was centrifuged again (12,000 × *g* for 20 min at 4°C), which resulted in three layers, top aqueous layer containing RNA was collected and mixed with equal volume of isopropanol and gently mixed by inverting the tubes. The mixture was kept at room temperature for 10 min and centrifuged at 12,000 × *g* for 15 min at 4°C. The supernatant was discarded followed by addition of 1 ml of 96% to 100% ethanol to the RNA pellet and centrifuged at 12,000 × *g* for 10 min at 4°C. The supernatant was discarded, and pellet was air dried. The pellet was dissolved in 20 μl of nuclease-free water and treated with DNAse I (Thermo-Scientific, USA) for removal of genomic DNA. Quality and quantity of RNA was assessed using Nanodrop (ND-1000, Thermo-Scientific, USA). RNA samples with 260/280 ratio of 1.8 to 2.0 were considered for cDNA synthesis. The cDNA was synthesized for 500 mg of total RNA using RevertAid First Strand cDNA Synthesis kit (Thermo-Scientific, USA) as per the manufacturer's protocol.

The primer sequences and corresponding annealing temperatures for select genes are given in Table 1. Annealing temperature for the primers was optimized for each gene using gradient PCR. Quantitative real-time PCR was performed as follows, the reaction mixture was prepared using 5 μl of SYBR green master mix (Thermo-Scientific, USA), 0.5 μl of each forward and reverse primer (10 pmol/μl), 2.0 μl of cDNA template, and 2.0 μl of nuclease free water to make up the final volume to 10 μl. The thermal cycling condition maintained in the qPCR experiment were 95°C for 20 s, 40 cycles of 95°C for 15 s, X°C (Table 1) for 15 s, and 72°C for 45 s followed by a dissociation protocol 95°C for 15 s plus 60°C for 15 s with increment of 0.3°C per minute. Each run was performed in duplicate and included a non-template control. The qPCR expression data for each gene were extracted in the form of quantification cycle (Ct), and data was subjected for subsequent analysis. The appropriate size of amplified products was ensured by 2% agarose gel electrophoresis (Supplementary Figure 2). *GAPDH* was used as internal control gene, whereas high- and low-fertile bull spermatozoa sample was taken as the control and target, respectively. Relative expression was calculated using the 2^−Δ*ΔCT*^ method given by Schmittgen and Livak (27).

**Table 1:** Primer details used for real-time quantification: primer 3 software (NCBI Primer designing tool).

**Sl. no**.	**Genes**	**Primer sequence**	**Product size**	**Annealing temperature**	**Accession number**
1.	TFAP2C	FP-ATGAAGAGGACTGCGAGGATCG	131 bp	64°C	NM_001075509
		RP-GTATTCGGCGACTCCGGTATG			
2.	YBX1	GTTGAAGGAGAAAAGGGTGCG	139 bp	60°C	NM_174815
		GCTGGTAATTGCGTGGAGGA			
3.	ORAI3	FP-CTTCCAAGCCGTCCTCTGTT	189 bp	60°C	NM_001193202
		RP-AGGAGCGGTAGAAATGCAGG			
4.	GAPDH	FP-CTGAGGACCAGGTTGTCTCCTG	141 bp	60°C	NM_001034034.1
		RP-CCCTGTTGCTGTAGCCAAATTC			

### Statistical Analysis

For microarray data analysis, after normalizing the probe signal intensities, a fold change was recorded for each probe indicating the transcript abundance ratio/mRNA in both samples. For the statistical inferences in calculating differential expression, the high fertile sample was tagged as the control, while the low fertile as treated. Percentile shift normalization is a global normalization, where the locations of all the spot intensities in an array are adjusted. This normalization takes each column in the experiment independently and computes the percentile of the expression values, across all spots (where n has a range from 0 to 100, and *n* = 75 is the median). It subtracts this value from the expression value of each entity. Gene expression level analysis was done by *t*-test. The differentially expressed gene ID's were uploaded in DAVID Bioinformatics Resources 6.8 (Laboratory of Human Retrovirology and Immunoinformatics, USA) for gene functional annotation, clustering, and KEGG pathway analysis. Gene ontology of dysregulated genes was studied using PANTHER classification system (version 14.0, Southern California), and the result was obtained as suitable plots. PANTHER GO-slim functions were considered, and the test type was Fisher's exact with FDR multiple test correction. The transcripts were considered as dysregulated when the fold change was more than one (28, 29).

One-way ANOVA and Duncan *post hoc* test was performed to assess significant differences in sperm phenotypic attributes among the three categories of the bulls. Kruskal–Wallis one-way analysis of variance was performed to understand the differences in gene expressions among bulls with different fertility ratings (HF, MF, and LF). The difference was considered significant when *P* < 0.05. Statistical analysis was performed using SPSS version 20 (IBM, USA).

## Results

### Sperm Functional Attributes in Bulls With Different Fertility Ratings

**Figure 2:**
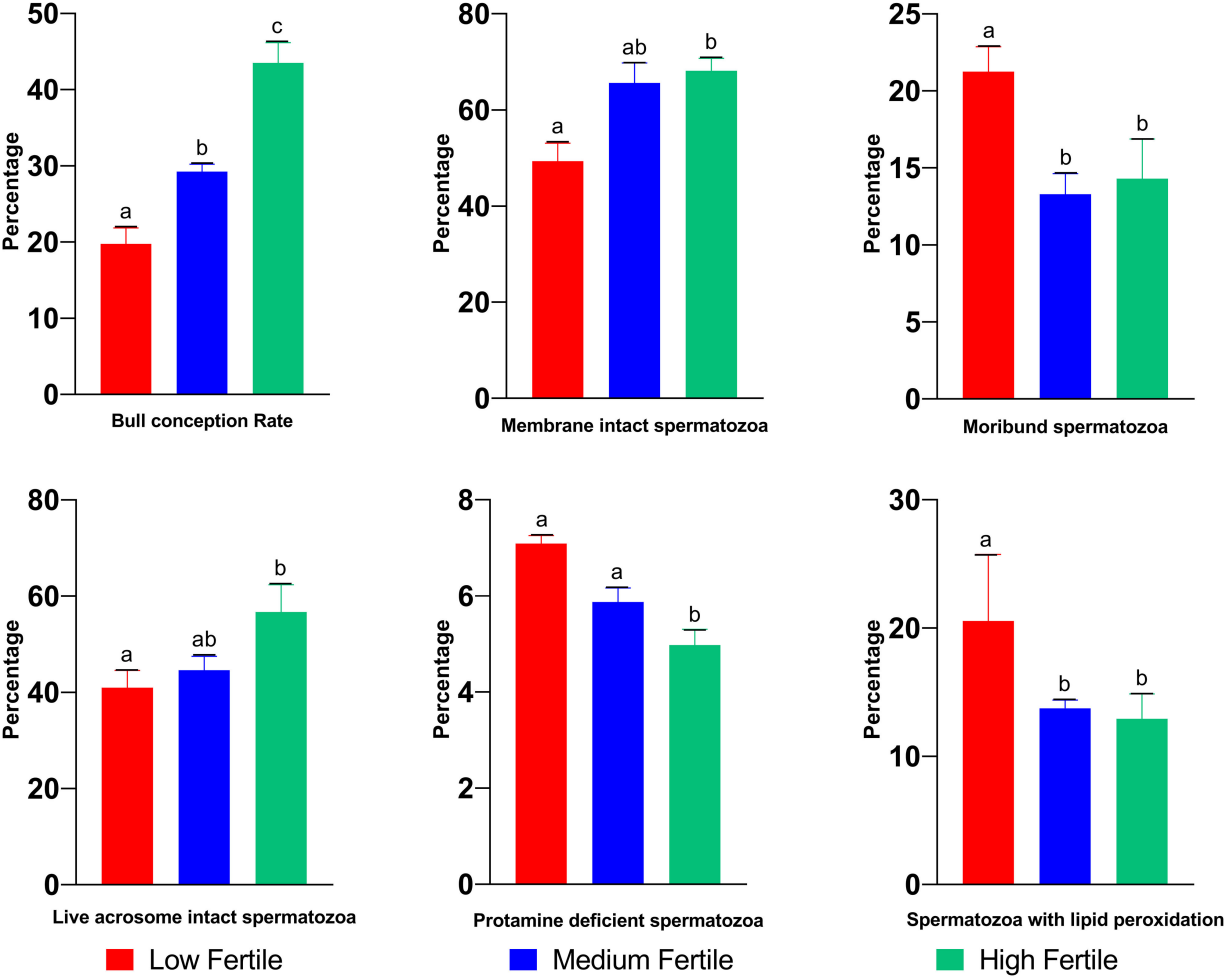
Sperm functional attributes of high-, medium-, and low-fertile buffalo bulls. Bars bearing different alphabets (a–c) significantly (*P* < 0.05) differ from each other within the parameter.

The mean conception rate of high, medium-, and low-fertile Murrah buffalo bulls was 19.2, 29.3, and 43.4%, respectively, and the differences among the three groups were significant (*P* < 0.05; Figure 2). Differences in sperm phenotypic attributes between high-, medium-, and low-fertile buffalo bulls are shown in the Figure 2. The proportion of progressive motile spermatozoa was higher (*P* < 0.05) in high-fertile bulls compared to medium and low-fertile bulls. While the proportion of membrane intact spermatozoa was significantly (*P* < 0.05) higher in high-fertile bulls, the proportion of moribund sperm population was significantly higher (*P* < 0.05) in low-fertile bulls. The proportion of live acrosome intact spermatozoa was significantly higher (*P* < 0.05) in high-fertile bulls compared to low-fertile bulls. On the other hand, the proportion of spermatozoa with protamine deficiency was higher in low-fertile bulls compared to either high- or medium-fertile bulls. The proportion of spermatozoa with lipid peroxidation was higher in low-fertile bulls compared to both medium and high-fertile bulls.

**Figure 3:**
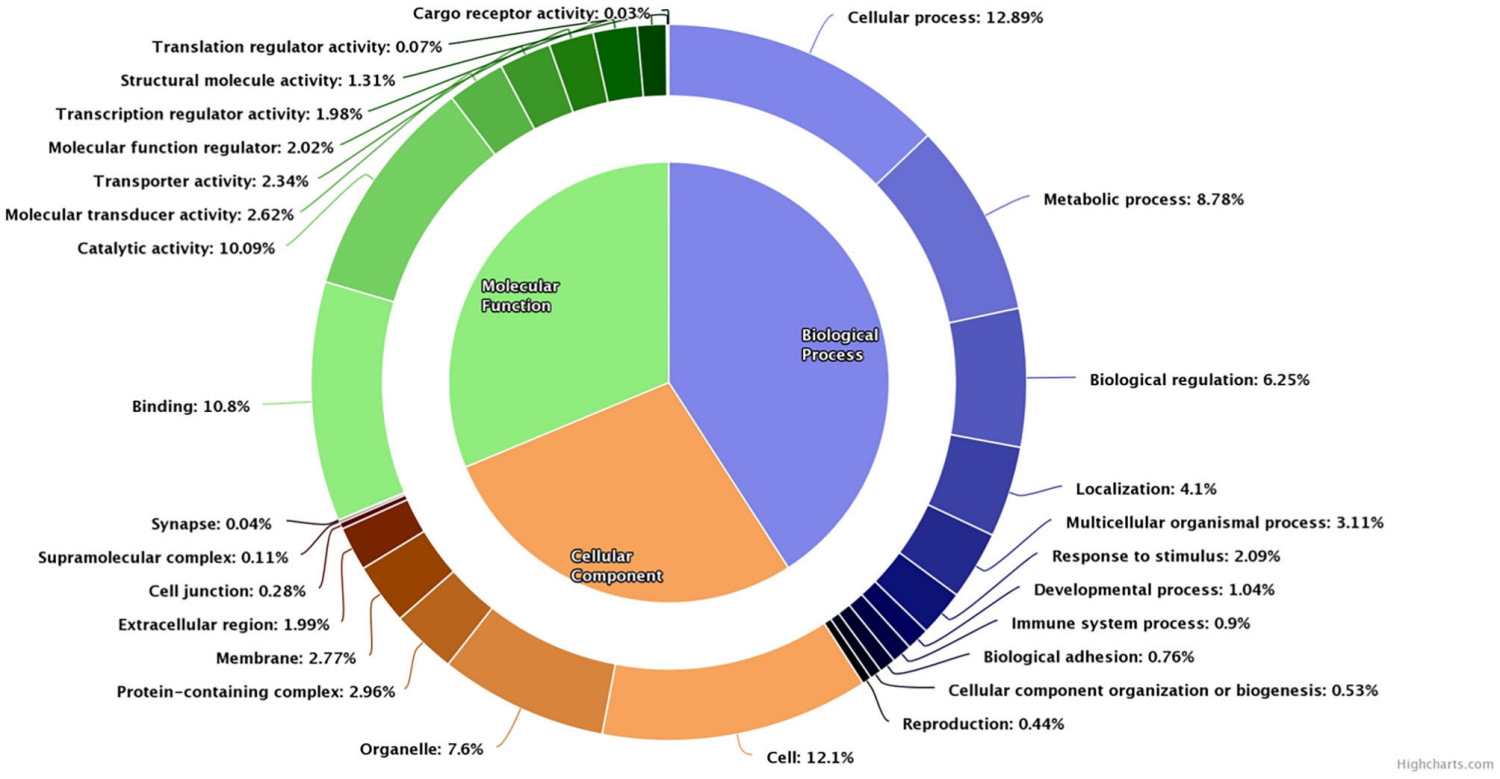
Pie donut diagram for gene ontology (biological process, molecular function, and cellular component) of total detected transcripts in low-fertile buffalo bull spermatozoa.

The array chip consisted of 51,282 number of probes designed against 32,430 genes. In buffalo spermatozoa, a total of 9,216 transcripts were detected. The gene ontology of the detected transcripts is depicted in Figure 3. Gene ontology analysis revealed that transcripts were associated with biological processes (8,672 transcripts), molecular function (6,630 transcripts), and cellular components (5,907 transcripts). Majority of the transcripts were involved in cellular processes (2,734 transcripts), metabolic process (1,862 transcripts), biological regulation (1,326 transcripts), and developmental processes (220 transcripts). Binding appeared as the predominant molecular function associated with the detected transcripts. Other molecular functions such as catalytic activity (2,140 transcripts), molecular transducer (556 transcripts), transporter activity (496 transcripts) and structural activity (278 transcripts) were also detected in buffalo spermatozoa. On the other hand, in cellular component, 2,566 transcripts were localized in sperm cell, 1,305 transcripts in intracellular, and 1,612 transcripts in cell organelle.

### Differentially Expressed Transcripts Between High- and Low-Fertile Buffalo Bull Spermatozoa

**Figure 4:**
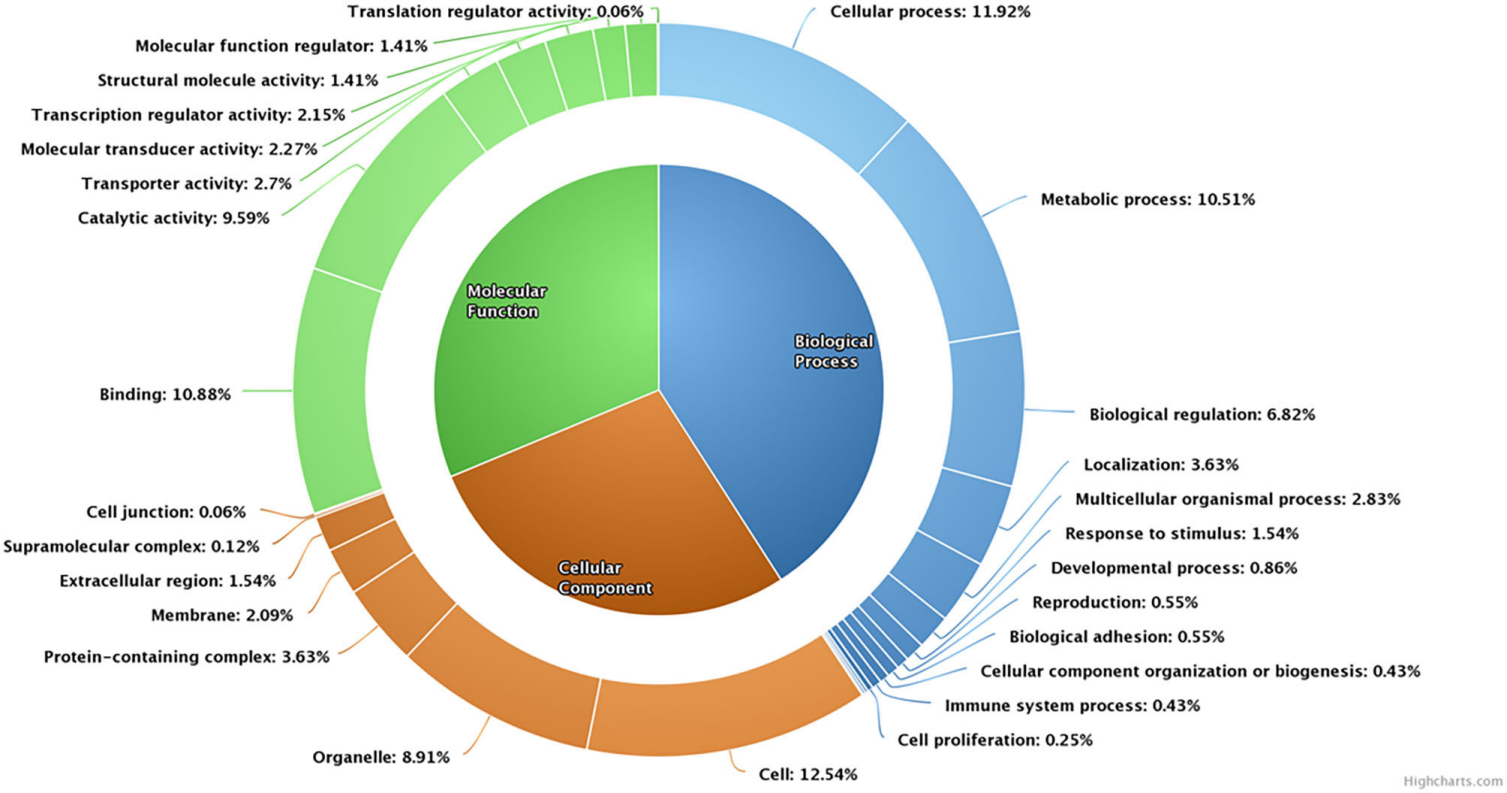
Pie donut diagram for gene ontology (biological process, molecular function, and cellular component) of dysregulated transcripts in low-fertile buffalo bull spermatozoa.

A total of 4,050 transcripts were differentially expressed between high- and low-fertile spermatozoa of buffalo bulls. Of which, 113 transcripts were upregulated, whereas 596 transcripts were downregulated in low-fertile buffalo bull spermatozoa. Dysregulated transcripts were classified based on their fold change (>1) and level of significance (i.e., *P* < 0.05). Additionally, PANTHER software was also used for gene ontology enrichment analysis, which revealed cellular process (11.29%), cell (12.54%), and binding (10.88%) as the most predominant GO terms in low-fertile buffalo bull spermatozoa (Figure 4).

**Figure 5:**
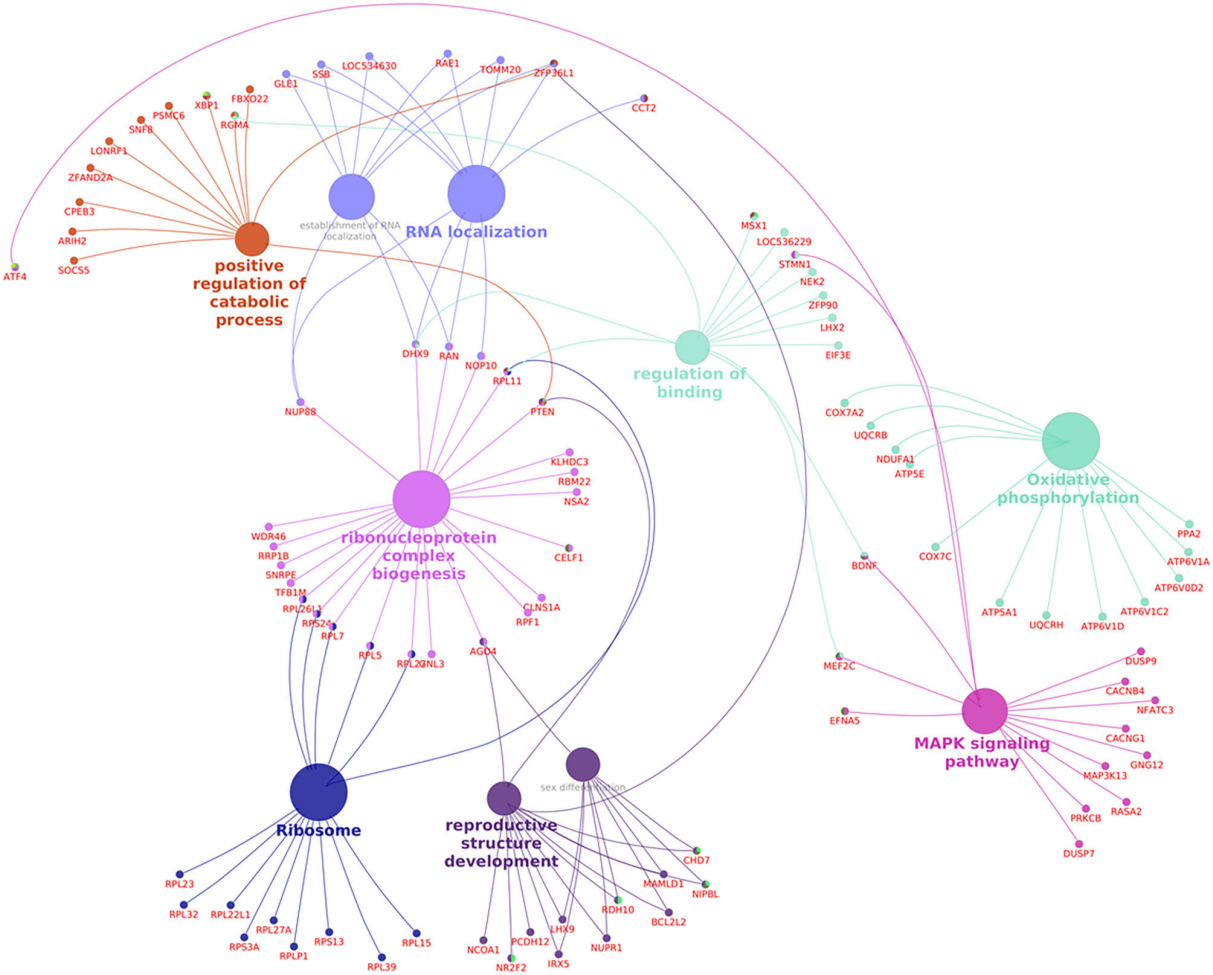
Pathway analysis and gene–gene interaction of differentially expressed transcripts in spermatozoa of low-fertile buffalo bulls generated using Cluego Plugin for cytoscape 3.7.1.

Pathway analysis and gene–gene interaction of the differentially expressed transcripts are shown in Figure 5. Differentially expressed transcripts were analyzed using the DAVID bioinformatic tool, which revealed 104 upregulated pathways and 272 downregulated pathways in low-fertile buffalo bull. The top 10 downregulated and upregulated pathways and associated genes in low-fertile bull spermatozoa are depicted in Tables 2 and 3, respectively. Among these pathways, MAPK signaling, ribosome and oxidative phosphorylation were found to be highly downregulated in low-fertile bulls with more number of gene counts (Supplementary Figures 3–5).

**Table 2:** Top 10 downregulated pathways in low-fertile buffalo bull spermatozoa.

**Pathway**	**Count**	**Genes involved**
Alzheimer's disease	28	*CDK5R1, APH1A, IDE, COX7B, COX7C, ATP5G1, COX5A, ATP5G3, NDUFB1, APP, NDUFS4, COX6B1, CHP, ATP5H, NDUFA4, NDUFA5, COX7A2, COX8A, LOC617654, CDK5, NAE1, COX6C, ATP2A2, UQCRH, GSK3B, ATP5A1, UQCRB, CALM1*
**MAPK signaling pathway**	28	*FGFR2, MEF2C, FGFR1, FGF18, HSPA1A, GNG12, CACNB4, BDNF, RASGRP3, SOS2, PAK1, CHP, HSPA8, RASA2, NLK, MAP2K4, CACNG6, TAOK3, MAPK10, PRKCB, ATF4, DUSP1, MAPK9, MAPK8, STMN1, MAP3K13, DUSP7, CD14, DUSP6*
**Ribosome**	27	*RPL19, RPL22L1, RPL38, RPS2, RPL39, LOC513245, RPL30, RPL32, RPS3A, RPL31, RPLP1, RPL3, RPL11, RPL5, RPL4, RPL10A, RPS23, RPS24, RPL35A, LOC783146, RPL27, RPS4X, RPL23, RPL13A, RPS12, RPS4Y1, RPS13, RPS10*
Pathways in cancer	27	*FGFR2, FGFR1, FGF18, PTEN, MMP1, PTK2, ITGAV, SOS2, RALB, NKX3-1, NOS2, FIGF, FN1, BMP4, PIK3CG, HSP90AA1, CTBP2, RUNX1T1, SKP2, RXRG, SMAD2, MAPK10, CTNNA3, RAD51, PRKCB, GSK3B, MAPK9, MAPK8*
Huntington's disease	25	*POLR2E, COX7B, COX7C, TBP, ATP5G1, COX5A, ATP5G3, NDUFB1, BDNF, NDUFS4, COX6B1, ATP5H, NDUFA4, NDUFA5, COX7A2, SLC25A4, SLC25A5, COX8A, PPARGC1A, COX6C, VDAC1, SP1, UQCRH, ATP5A1, UQCRB*
**Oxidative phosphorylation**	24	*NDUFA4, NDUFA5, COX11, COX7A2, COX8A, COX7B, COX7C, ATP5G1, COX5A, ATP5G3, ATP6V1D, NDUFB1, COX6C, ATP6V1C2, ATP6V1A, NDUFS4, UQCRH, ATP6V1E1, COX6B1, ATP5L, ATP5A1, ATP5H, ATP6V0D2, UQCRB*
**Regulation of actin cytoskeleton**	24	*FGFR2, PIK3CG, FGFR1, FGF18, VAV3, DIAPH1, MYL12A, GNG12, IQGAP1, VCL, ACTG1, ARPC1A, PAK7, PTK2, PFN2, TIAM1, ARPC2, GSN, ITGAV, SOS2, CYFIP2, PAK1, CD14, FN1*
Parkinson's disease	22	*NDUFA4, NDUFA5, COX7A2, SLC25A4, SLC25A5, COX8A, COX7B, COX7C, ATP5G1, COX5A, UBE2L3, ATP5G3, NDUFB1, VDAC1, COX6C, NDUFS4, UQCRH, SLC18A2, COX6B1, ATP5A1, ATP5H, UQCRB*
Focal adhesion	22	*PIK3CG, CAV1, FLT1, VAV3, DIAPH1, MYL12A, MAPK10, PTEN, VCL, PRKCB, ACTG1, PAK7, PTK2, FYN, GSK3B, ITGAV, SOS2, MAPK9, TNN, MAPK8, PAK1, FIGF, FN1*
Neuroactive ligand–receptor interaction	22	*F2RL2, GABRG1, CCKAR, GABRG3, GABRA2, DRD1, GLRB, RXFP1, GABRB1, DRD5, HTR4, S1PR3, ADRB2, S1PR1, P2RX6, GRIA2, P2RY2, MC4R, LOC616737, PRL, LOC782768, GRID1*

Pathways indicated in bold are reported to be related to bull spermatozoa. The roles of other pathways in buffalo bull spermatozoa are not known.

**Table 3:** Top 10 upregulated pathways in low-fertile buffalo bull spermatozoa.

**Pathway**	**Count**	**Genes involved**
**Cell adhesion molecules (CAMs)**	9	*MAG, CADM3, CLDN3, ITGB7, CLDN5, BOLA-DQB, ITGB2, BOLA-N, CD6*
Vascular smooth muscle contraction	8	*RAMP2, ARHGEF1, ADORA2A, ADCY6, CALM3, PLA2G2D1, ITPR1, ARHGEF11*
Endocytosis	8	*GIT1, ADRB3, RAB11FIP3, SNF8, VPS4A, BOLA-N, SH3GL1, VPS25*
**MAPK signaling pathway**	8	*RELB, MAPK11, MAPK8, CACNG2, PLA2G2D1, CACNG1, DUSP9, MAP2K6*
Hypertrophic cardiomyopathy (HCM)	7	*ITGB7, MYBPC3, ITGB4, PRKAA2, CACNG2, CACNG1, TPM1*
Dilated cardiomyopathy	7	*ITGB7, ADCY6, MYBPC3, ITGB4, CACNG2, CACNG1, TPM1*
**GnRH signaling pathway**	7	*ADCY6, CALM3, MAPK11, MAPK8, PLA2G2D1, MAP2K6, ITPR1*
Leukocyte transendothelial migration	7	*CYBA, GNAI2, CLDN3, CLDN5, MAPK11, ITGB2, PXN*
Neurotrophin signaling pathway	7	*ZNF274, NFKBIA, CALM3, SH2B3, MAPK11, MAPK8, SH2B1*
Wnt signaling pathway	7	*CTBP2, NKD2, CREBBP, FZD1, FRAT1, MAPK8, SIAH1*

Pathways indicated in bold are reported to be related to bull spermatozoa. The roles of other pathways in buffalo bull spermatozoa are not known.

**Figure 6:**
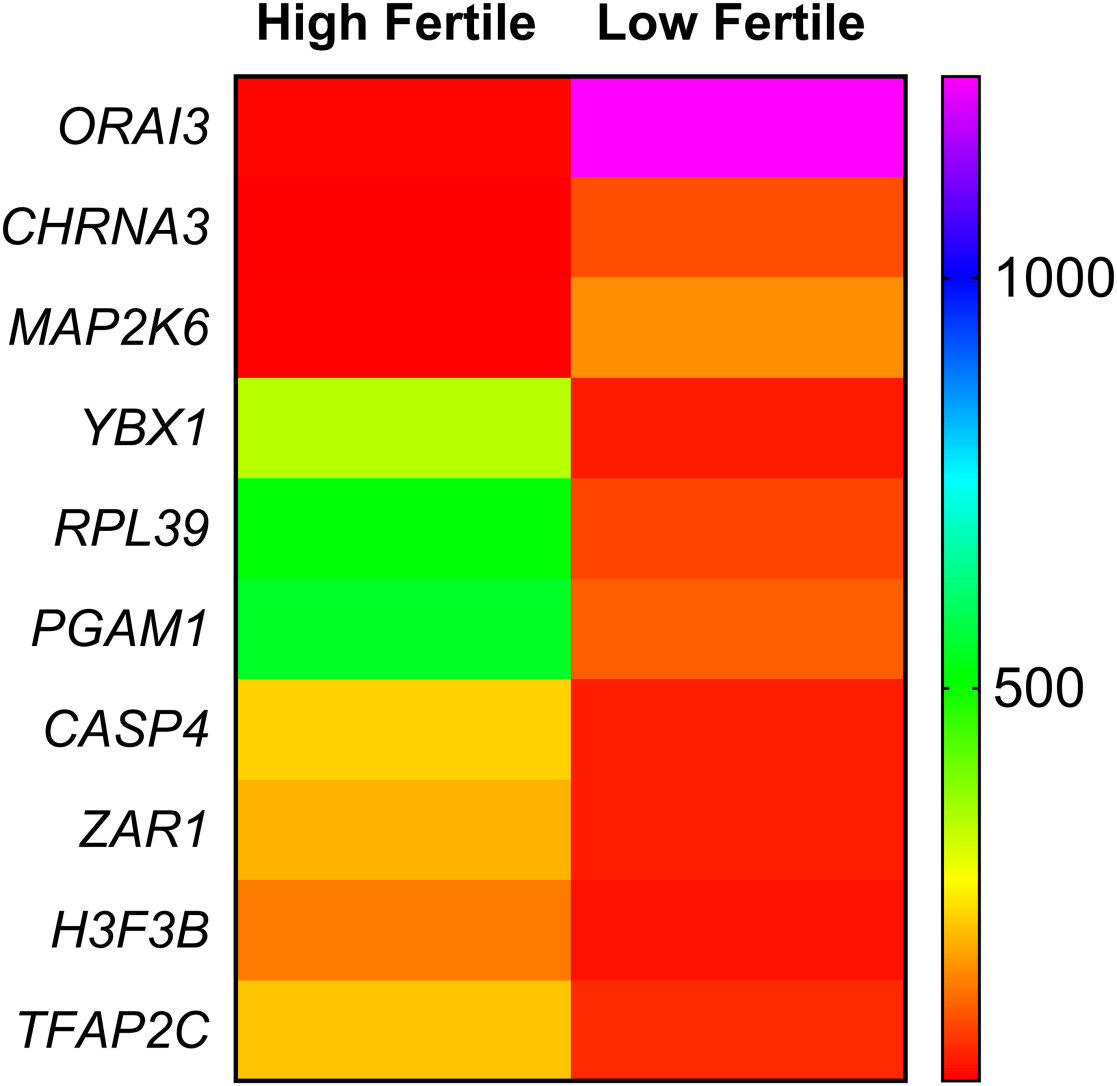
Heat map depicting relative gene expression of 10 differentially expressed genes related to sperm function. Each row indicates a single gene, and each column indicates two sample groups (high fertile and low fertile). The difference in color in any row of heat map is indicating a differential expression of that particular gene between high- and low-fertile buffalo bull spermatozoa.

Among the dysregulated genes, based on the earlier reports, we identified 10 genes having a role in sperm functional attributes (*YBX1*, RPL39, PGAM1, *CASP4, TFAP2C, H3F3B, ZAR1, CHRNA3, MAP2K6*, and *ORAI3)* and found that their expression were significantly altered in low-fertile bulls (Figure 6). The reported roles of these genes in sperm functional attributes and embryonic development are given in Table 4. Among these transcripts, *YBX1*, RPL39, PGAM1, *CASP4, TFAP2C, H3F3B*, and *ZAR1* were downregulated, while *CHRNA3, MAP2K6*, and *ORAI3* were upregulated in low-fertile buffalo bull spermatozoa. The gene *YBX1* was the most downregulated (−2.78-fold), and *ORAI3* was the most upregulated (3.71-fold) gene in low-fertile buffalo bull spermatozoa.

**Table 4:** Reported function of 10 fertility-related differentially expressed genes in low-fertile buffalo bull spermatozoa.

**Gene name**	**Fold change**	**Function**	**Reference**
*YBX1*	−2.78	Role in basic cellular functions. YB-1 has critical role for the survival of mid-to-late-stage mice embryos	(30)
*RPL39*	−2.50	Predominant expression in testis, expressed during spermatogenesis just before the rapid production of proteins that are required for sperm differentiation and maturation	(31)
*PGAM1*	−2.23	PGAM1 correlates with spermatogenic dysfunction and affects the function of cell proliferation, apoptosis, and migration, expression of PGAM1 might be correlated with hypospermatogenesis	(32)
*CASP4*	−2.09	Apoptotic gene expression in spermatozoa	(33)
*TFAP2C*	−1.73	Trophectoderm development in mice	(34)
*H3F3B*	−1.76	Important in germ cell development, regulating oogenesis, spermatogenesis, and fertilization in mice	(35)
*ZAR1*	−1.84	Evolutionarily conserved gene in vertebrates, essential for embryonic development in mice	(36)
*CHRNA3*	2.38	Initiation of AR by activating an a7 subunit-containing CHRNA3 initiated by using purified recombinant human ZP protein (rhZP3) and thus potentially has a role in the initiation of the AR by intact ZP *in vivo*	(37)
*MAP2K6*	2.32	Positively correlated with sperm motility, germ cell development, germ cell apoptosis, acquisition of motility in the epididymis, sperm capacitation, and acrosome reaction	(38)
*ORAI3*	3.71	Oocyte activation by transient calcium ion oscillations	(39)

### Real-Time Expression Analysis of *YBX1, ORAI3, TFAP2C* Genes in Spermatozoa From Buffalo Bulls With Different Fertility Ratings

**Figure 7:**
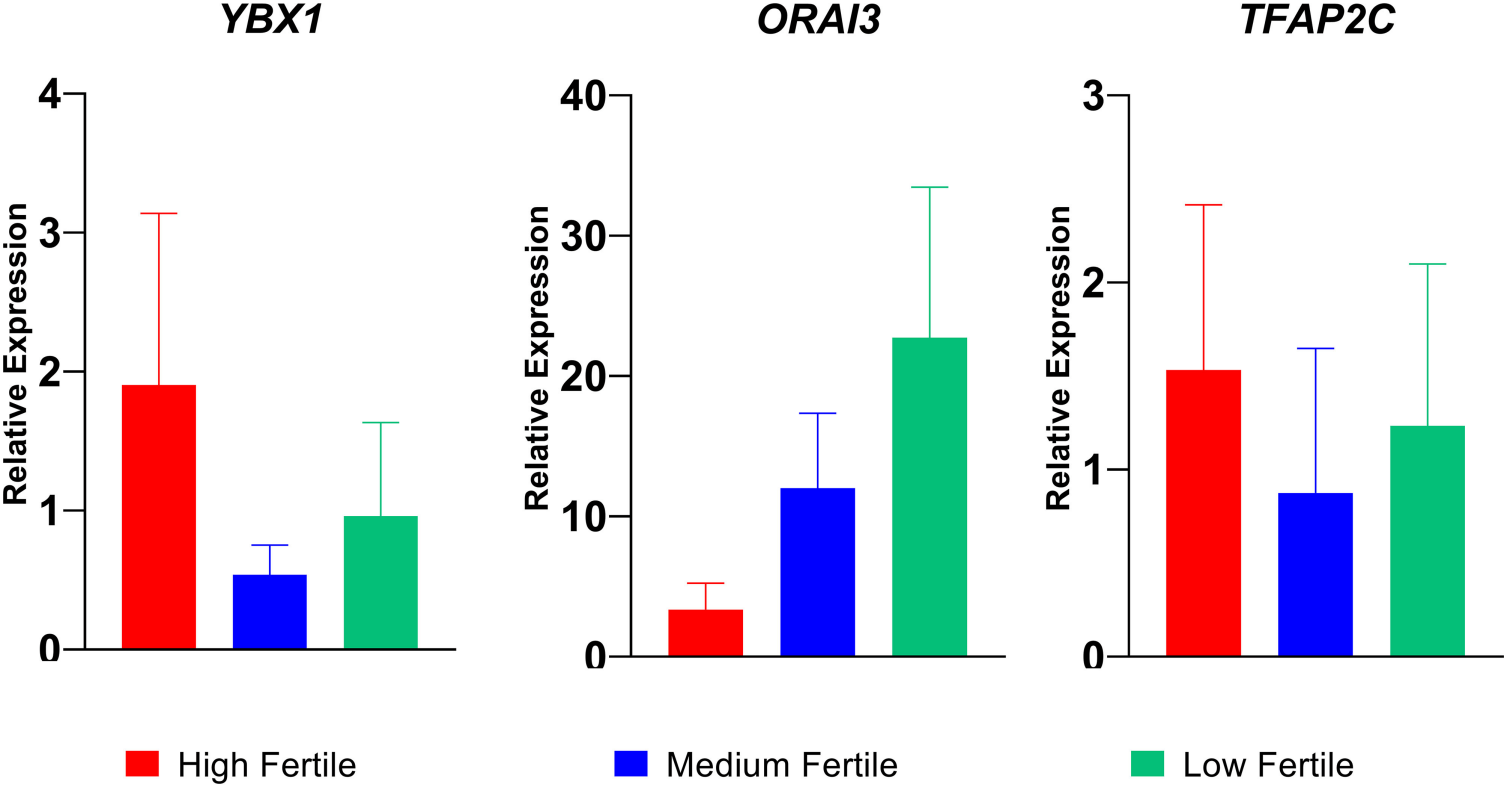
Relative expression of YBX1, ORAI3, and TFAP2C genes in high-, medium-, and low-fertile bull spermatozoa analyzed by real time qPCR.

Among the differentially regulated genes, we selected three dysregulated genes (*YBX1, TFAP2C*, and *ORAI3*) for validation in nine buffalo bulls (three each in high-, medium- and low-fertile category). The relative expressions of transcripts are given in Figure 7. We found that the sperm expression of ORAI 3 gene was higher and the expression of *YBX1* and *TFAP2C* genes were lower in low-fertile bulls compared to high-fertile bulls.

## Discussion

Recently, several studies have been conducted to understand the phenotypic and molecular differences between high- and low-fertile spermatozoa (4, 14, 40, 41). Since the molecular details of buffalo spermatozoa are not known, in the present study, we investigated the sperm functional differences and transcriptomic profile in relation to field fertility. We report here that sperm functional attributes were altered and transcripts involved in MAPK signaling, ribosome pathway, and oxidative phosphorylation were dysregulated in low-fertile bull spermatozoa.

In artificial breeding, it is well-understood that sperm must possess some functionalities for successful fertilization of oocyte, which include, but not restricted to, motility to reach the site of fertilization (42) functional membrane integrity to establish sperm–oviduct reservoir (4, 5, 43) and sperm–oocyte binding (44) and intact non-reacted acrosome for sperm penetration into the oocyte (5). Additionally, it has been reported that sperm DNA integrity (45) and status of lipid peroxidation (4, 46) are also related to male fertility. In the present study, we observed that sperm motility, membrane, and acrosomal integrity were significantly higher, while the status of protamination and lipid peroxidation were significantly lower in high- compared to low-fertile buffalo bulls. Earlier reports indicate that sperm motility had a positive relationship with fertility (47, 48), while the proportion of spermatozoa with altered functional membrane integrity had a negative correlation with fertility (49). The higher proportion of membrane compromised spermatozoa in low-fertile buffalo bull semen compared to high-fertile buffalo indicates reduced capacity of spermatozoa from low-fertile buffalo bulls to withstand extraneous factors. Similarly, higher proportion of moribund spermatozoa in a semen sample is undesirable as the dying spermatozoa are a potent source of reactive oxygen species, which negatively affects the functionality of normal spermatozoa (50). Apart from this, the importance of spermatozoa with functionally intact acrosome to be realized as the oocyte recognizes and embraces live acrosome intact spermatozoa for fertilization by a yet to be known mechanism, and the presence of low proportion of live intact acrosome spermatozoa in low-fertile bull spermatozoa compared to high-fertile bull spermatozoa indicates that less number of spermatozoa with functionally intact acrosome will reach the site of fertilization resulting in reduced chances of successful fertilization. Our observations on sperm protamination status are in agreement to earlier reports (51, 52). It is a proven fact that any aberration in sperm DNA protamination process could lead to an anomaly in packaging process of nucleus and eventually influences sperm morphology and negatively correlated with bull fertility (53). The trend in buffalo sperm lipid peroxidation status observed in our study is in line with the earlier reports (54, 55). Since buffalo spermatozoa contain higher amounts of poly unsaturated fatty acids, the susceptibility to oxidative damage also increases. Further, it was shown that bulls with high sperm membrane lipid peroxidation were more likely to have a high DNA fragmentation and less plasma membrane integrity (56). Therefore, these functional alterations in spermatozoa might be responsible for reduced fertility in low-fertile buffalo bulls.

We found that a total of 4,050 transcripts were differentially expressed between high- and low-fertile buffalo bull spermatozoa. Assessment of differential expression by fold change and level of significance indicated that a total of 709 genes were dysregulated. The dysregulated genes were mainly involved in cellular processes, binding activities, metabolic processes, and catalytic activities related to sperm function (sperm motility, capacitation, and acrosome reaction), fertilization (sperm–oocyte binding and zygote formation), and early embryonic development (maternal zygotic transition and early embryonic growth). Our observations are in agreement with the earlier studies who also observed the presence of transcripts, in spermatozoa, having significant role in sperm functional attributes, fertilization, and embryonic development (14, 57, 58). The pathway enrichment analysis of dysregulated genes indicated that MAPK signaling, ribosome pathway, and oxidative phosphorylation were found to be dysregulated in low-fertile buffalo bulls.

We observed that 28 genes associated with MAPK signaling pathway are downregulated in low-fertile buffalo bull spermatozoa. MAPKs are involved in the regulation of transcription in the testis and also regulate sperm motility, hyperactivation, and acrosome reaction (59), whereas its inhibition is required for resumption of meiosis in the oocyte, which triggers the formation of pronuclei in the process of fertilization (60). It has been reported that certain members of the MAPK family have a crucial influence on spermatozoa physiology, from motility to morphology, and any alterations in this pathway might affect sperm fertilizing ability. In line with these observations, we observed that the sperm motility was significantly lower in low-fertile bulls compared to high-fertile bulls. Oxidative phosphorylation is an important pathway of energy production in mammalian spermatozoa (61–65). We observed that 24 genes associated with the oxidative phosphorylation pathway were downregulated in low-fertile bull spermatozoa. A large amount of energy is required for sperm flagellar motion for traversing through barriers in the female reproductive tract and for hyperactivated motility to reach the site of fertilization (66). Apart from this, oxidative phosphorylation also has an intriguing role in acrosome reaction, which is a prerequisite for fertilization (67), and in this direction, in the present study, we observed higher proportion of pre-mature acrosome reacted spermatozoa in low-fertile bulls compared to high-fertile bulls. Since the members of the MAPK family and oxidative phosphorylation pathway are crucial to the proper functioning of signaling cascades that regulate sperm functions, downregulation of a large number of genes regulating both these pathways in low-fertile bull spermatozoa, as observed in the current study, might explain, at least partly, the reason for reduced fertility in these bulls.

Our study identified 27 downregulated transcripts associated with ribosome pathway. The ribosomal protein-encoding genes have been reported in mammals, and four of the ribosomal protein-encoding genes are present on the X chromosome (66) with their increased expression in testis during spermatogenesis just before rapid production of proteins that have a role in sperm differentiation and maturation (33). Accumulating evidences on ribosomal proteins has shown that some of the proteins encoded by ribosomal genes when underexpressed contribute to prostatic tumor formation in human males (68). Not only in males but reports show that some of the ribosomal transcripts are highly expressed in various tumor conditions (i.e., cervical tumor, ovarian tumor) in human females (69). Unfortunately, such information is not available in livestock, but with the available information on other species, it can be hypothesized that some of the ribosomal proteins may have a crucial role in sperm maturation and differentiation, and their downregulation may lead to impaired spermatozoon maturation resulting in reduction in semen quality in low-fertile buffalo bulls.

We observed that several gene-related intracellular calcium regulation, sperm functions, and embryonic development were differentially expressed between high- and low-fertile bulls. Among these genes, based on the earlier reports, we identified 10 genes having a role in sperm functional attributes (*YBX1*, RPL39, PGAM1, *CASP4, TFAP2C, H3F3B, ZAR1, CHRNA3, MAP2K6*, and *ORAI3)* and found that their expression was significantly altered in low-fertile bulls. Since sperm functionalities were found to be altered in spermatozoa of low-fertile bulls, we assessed relative expression of selected three genes using qPCR as validation of the findings. It was observed that the transcriptional abundance of ORAI3 gene was consistently higher in low-fertile bulls. ORAI proteins form the calcium-receptor release-activated channels in sperm plasma membrane (70) and are involved in calcium oscillations, which are essential for fertilization (71). However, upregulation of these genes in low-fertile bulls may induce early capacitation, acrosome reaction, and negatively affect the fertilization process. The other gene YBX-1, downregulated in low-fertile bull spermatozoa, plays an important role in transcription, regulation of cell cycle, and DNA repair (72, 73). The molecule CASP4 is said to have a role in apoptosis and was highly expressed in high-fertile men (33), while TFAP2C was found to be expressed in trophectoderm of blastocyst and have an intriguing role in placentation (74, 75). H3F3B is involved in nuclear reprogramming and transcription of embryonic genes (76), while ZAR1 has a role in cleavage and maternal zygotic transition (77, 78). RPL39 and PGAM1 have a role in sperm maturational process and apoptosis, respectively (32). Collectively, all these genes are involved in intracellular calcium regulation, cleavage, and early embryonic development, and downregulation of these genes might be a reason for reduced fertility of spermatozoa from low-fertile buffalo bulls.

## Conclusions

In gist, the present study is the first to report the transcriptomic profile of buffalo bull spermatozoa and to identify the differences in sperm transcriptomic profiles between high- and low-fertile buffalo bulls. It is inferred that genes related to sperm functional attributes, intracellular calcium regulation, and embryonic development were downregulated in low-fertile buffalo bull spermatozoa. Pathways associated with MAPK signaling, ribosome, and oxidative phosphorylation were significantly dysregulated in low-fertile bull spermatozoa. Further detailed studies on the identified differentially expressed pathways at protein level would expand our understanding the sperm quality differences between high- and low-fertile bulls and also to identify suitable molecules for buffalo bull fertility prediction.

## Data Availability Statement

The datasets presented in this study can be found in online repositories. The names of the repository/repositories can be found with the accession number GSE154517 at: https://www.ncbi.nlm.nih.gov/geo.

## Ethics Statement

The animal study was reviewed and approved by Institute Animal Ethics Committee.

## Author Contributions

NP investigated, visualized, and wrote the original draft. AK conceptualized the study, contributed to the project administration, provided the resources, wrote, reviewed, and edited the manuscript, and supervised the study. MD contributed to the methodology, and wrote, reviewed, and edited the manuscript. PN contributed to the data curation and formal analysis, and wrote, reviewed, and edited the manuscript. PG and CK contributed to the methodology and investigation. AS contributed to the methodology and investigation, and wrote, reviewed, and edited the manuscript. SS wrote, reviewed, and edited the manuscript. TD contributed to the formal analysis, and wrote, reviewed, and edited the manuscript. All authors contributed to the article and approved the submitted version.

## Conflict of Interest

The authors declare that the research was conducted in the absence of any commercial or financial relationships that could be construed as a potential conflict of interest.

## Supplementary Material

Supplementary Files The detailed list of differentially expressed transcripts between high- and low-fertile buffalo bull spermatozoa.

Supplementary Figure 1 In vivo conception rates of buffalo breeding bulls used in the study. Green colour bars indicate low-fertile bulls while red colour bars indicate high-fertile bulls used for transcriptomic analysis of spermatozoa.

Supplementary Figure 2 Quantitative real-time PCR products for various candidate genes in buffalo spermatozoa: M, 50 bp DNA marker; 1, ORAI3 (189 bp); 3, YBX1 (139 bp); 5, TFAP2C (131 bp); 7, GAPDH (141 bp); 2,4,6,8, NTC.

Supplementary Figure 3 Dysregulated transcripts affecting MAPK signalling pathway in low fertile buffalo bull spermatozoa (Red box indicate downregulated transcripts in low fertile buffalo bull spermatozoa).

Supplementary Figure 4 Dysregulated transcripts affecting ribosome pathway in low fertile buffalo bull spermatozoa (Red box indicate downregulated transcripts in low fertile buffalo bull spermatozoa).

Supplementary Figure 5 Dysregulated transcripts affecting oxidative phosphorylation pathway in low fertile buffalo bull spermatozoa (Red box indicate downregulated transcripts in low fertile buffalo bull spermatozoa).
