# Supplementary figures and images for: Transcriptomic Profiling of Buffalo Spermatozoa Reveals Dysregulation of Functionally Relevant mRNAs in Low-Fertile Bulls
Source: Front Vet Sci. 2021 Jan 11;7:609518. doi: 10.3389/fvets.2020.609518 (PMC7829312; doi:10.3389/fvets.2020.609518)

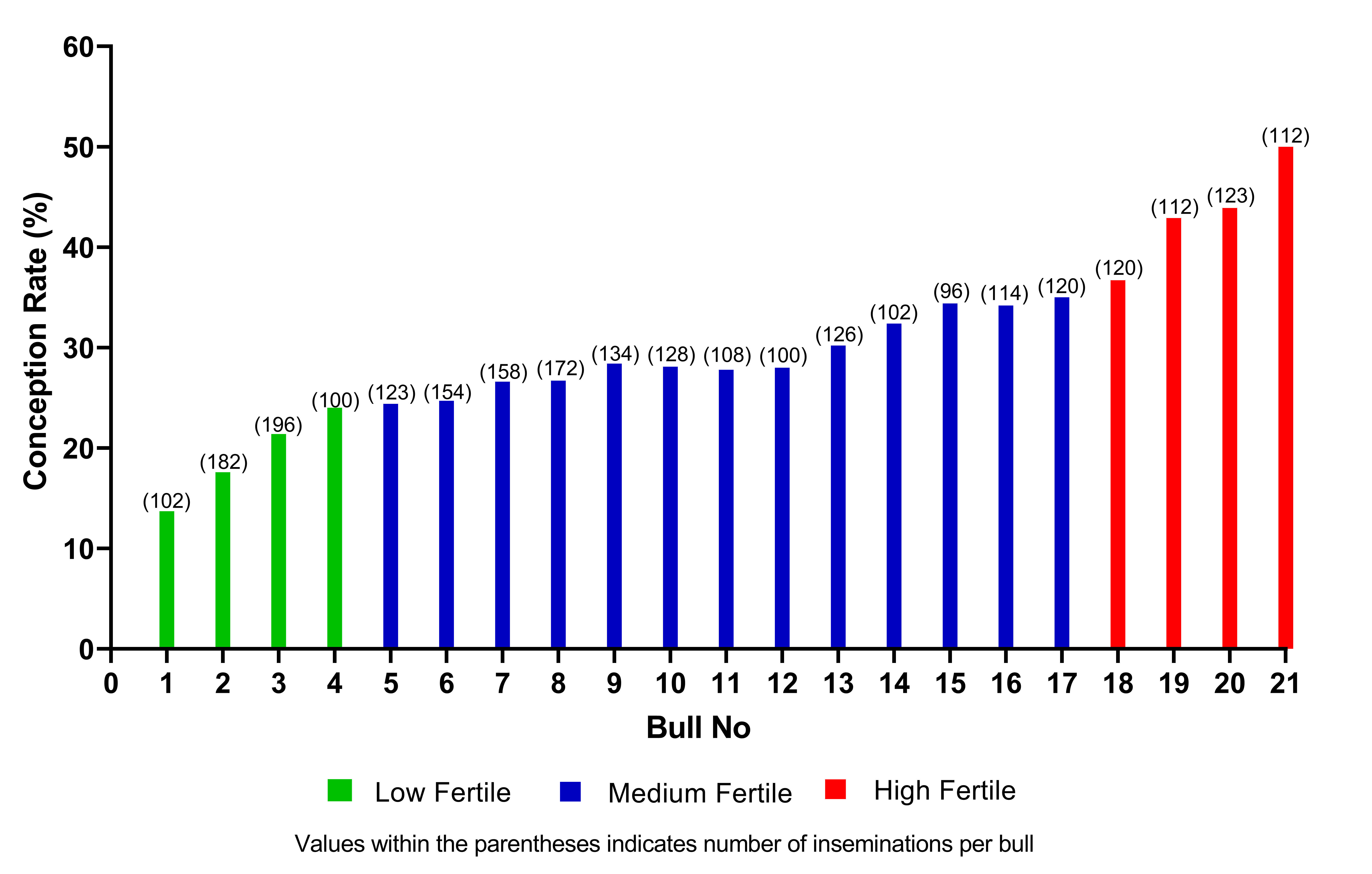

Supplement: Supplementary Figure 1 — In vivo conception rates of buffalo breeding bulls used in the study. Green colour bars indicate low-fertile bulls while red colour bars indicate high-fertile bulls used for transcriptomic analysis of spermatozoa. [file Image_1.TIF]

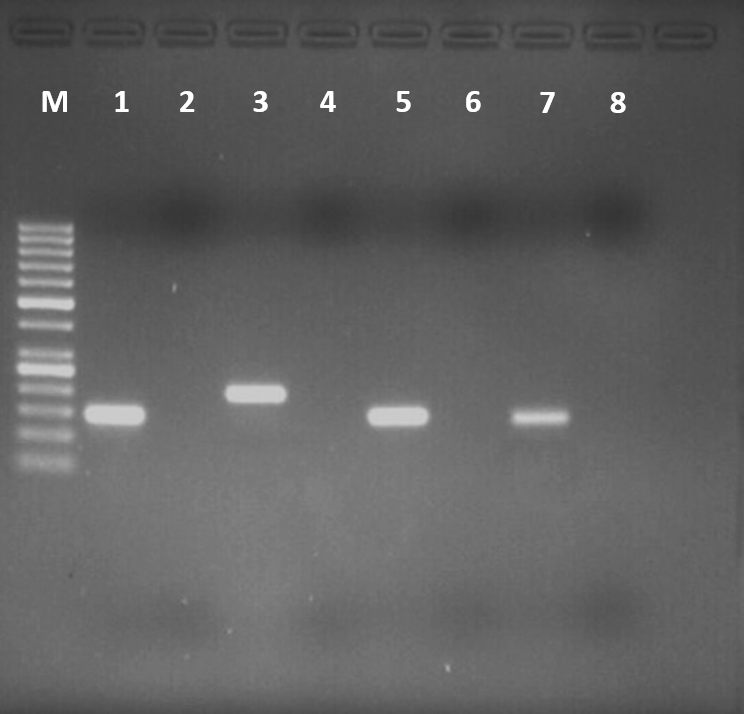

Supplement: Supplementary Figure 2 — Quantitative real-time PCR products for various candidate genes in buffalo spermatozoa: M, 50 bp DNA marker; 1, ORAI3 (189 bp); 3, YBX1 (139 bp); 5, TFAP2C (131 bp); 7, GAPDH (141 bp); 2,4,6,8, NTC. [file Image_2.TIF]

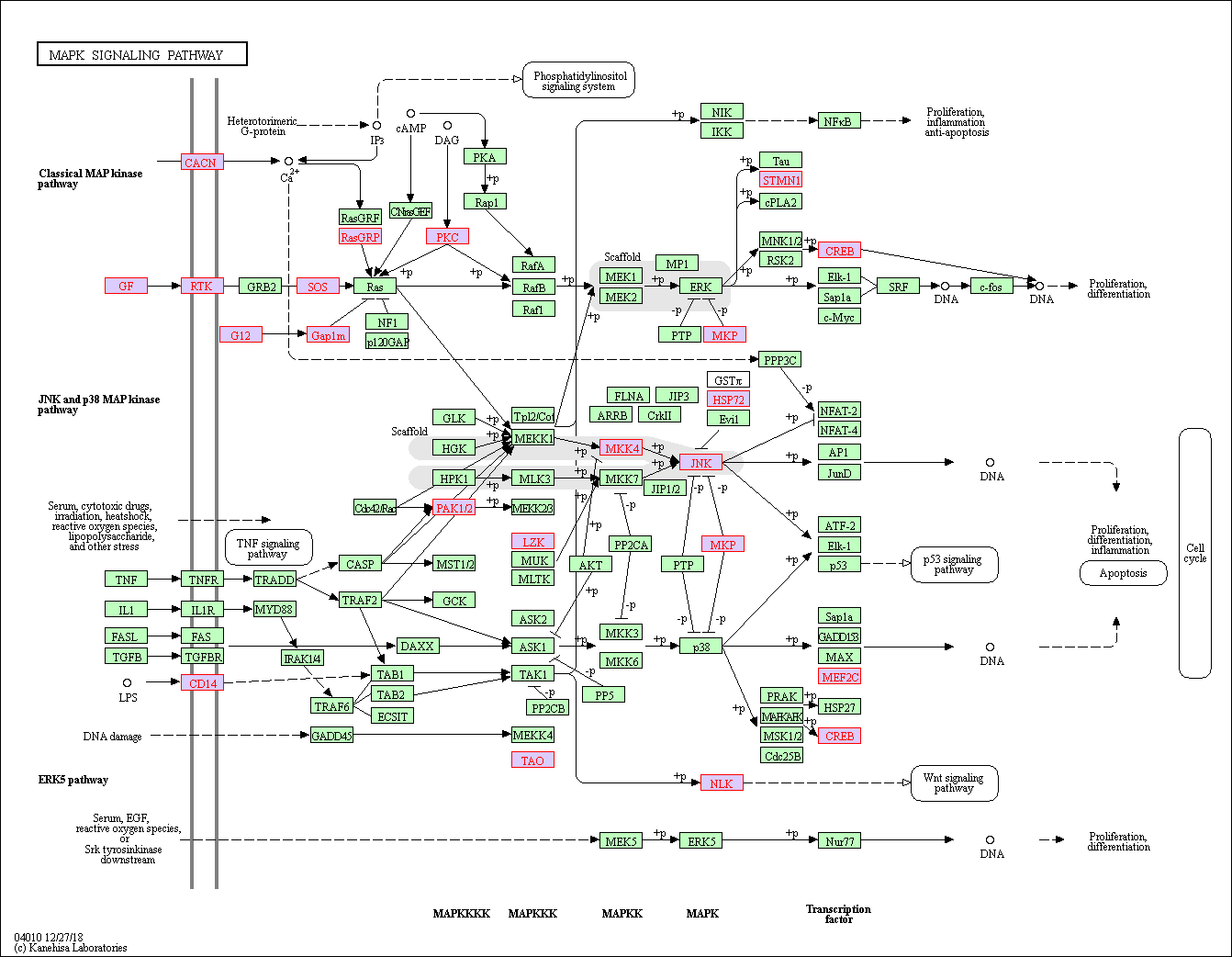

Supplement: Supplementary Figure 3 — Dysregulated transcripts affecting MAPK signalling pathway in low fertile buffalo bull spermatozoa (Red box indicate downregulated transcripts in low fertile buffalo bull spermatozoa). [file Image_3.TIFF]

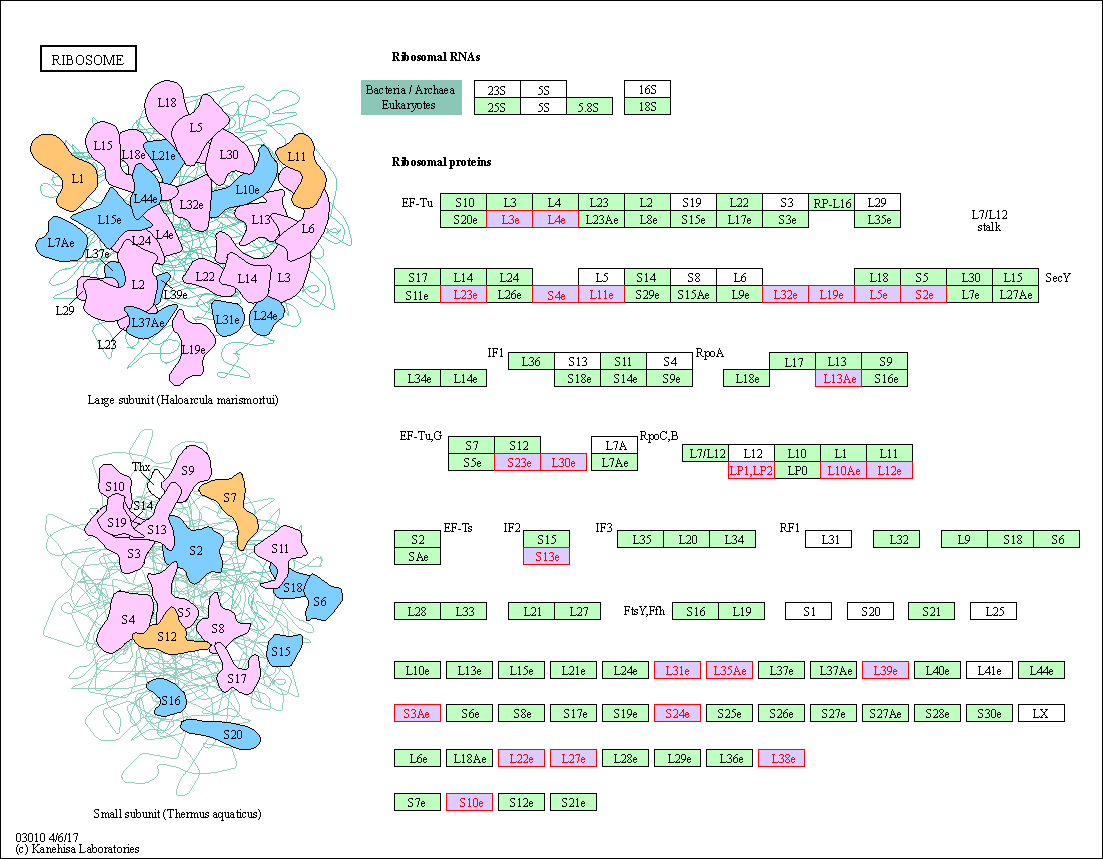

Supplement: Supplementary Figure 4 — Dysregulated transcripts affecting ribosome pathway in low fertile buffalo bull spermatozoa (Red box indicate downregulated transcripts in low fertile buffalo bull spermatozoa). [file Image_4.TIFF]

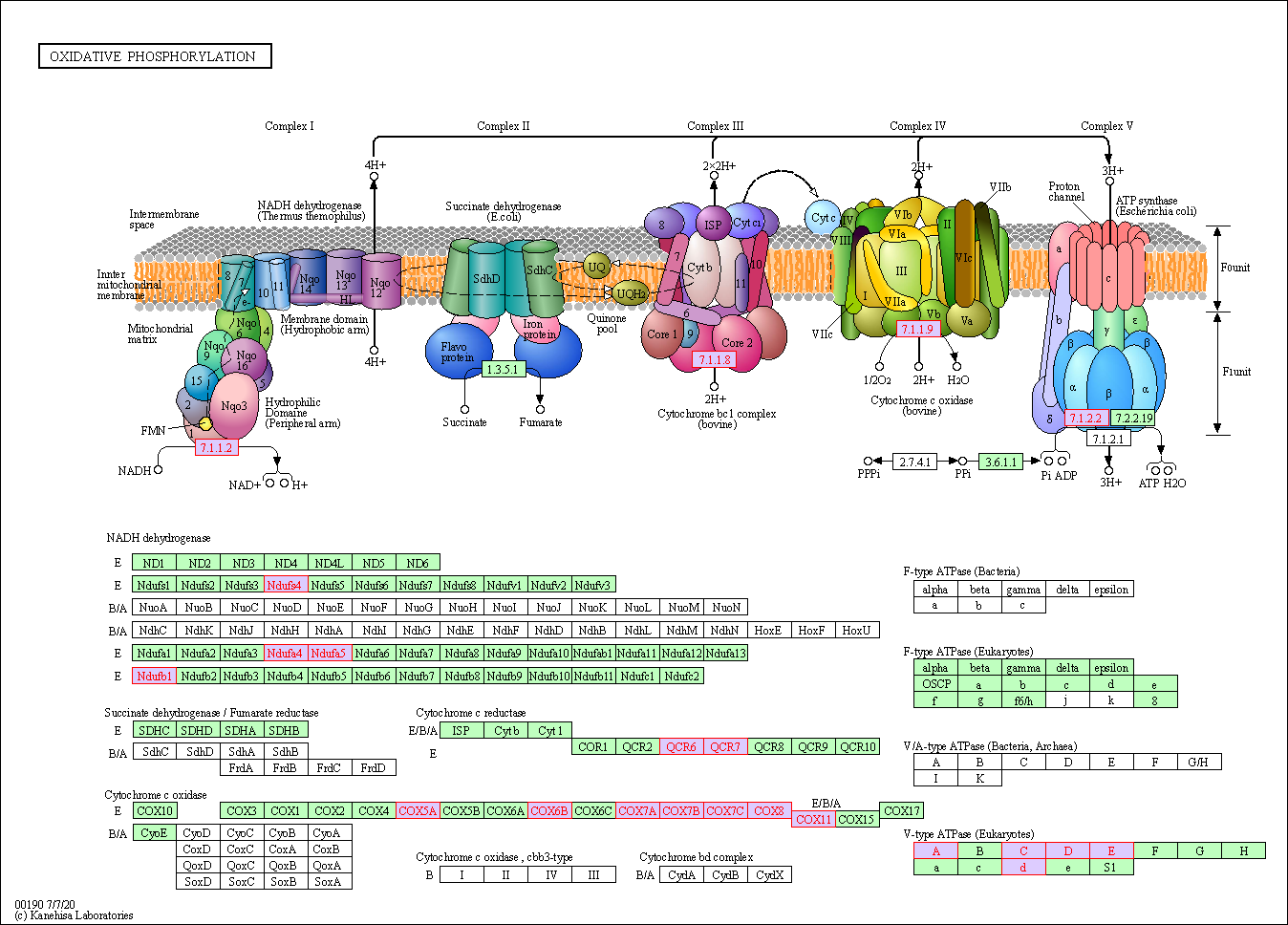

Supplement: Supplementary Figure 5 — Dysregulated transcripts affecting oxidative phosphorylation pathway in low fertile buffalo bull spermatozoa (Red box indicate downregulated transcripts in low fertile buffalo bull spermatozoa). [file Image_5.TIFF]
